# Supplementary material for: Epigenomic diversity of cortical projection neurons in the mouse brain
Source: Nature. 2021 Oct 6;598(7879):167–73. doi: 10.1038/s41586-021-03223-w (PMC8494636; doi:10.1038/s41586-021-03223-w)

nature portfolio

https://doi.org/10.1038/s41586-021-03223-w

Supplementary information

Epigenomic diversity of cortical projection neurons in the mouse brain

In the format provided by the
authors and unedited

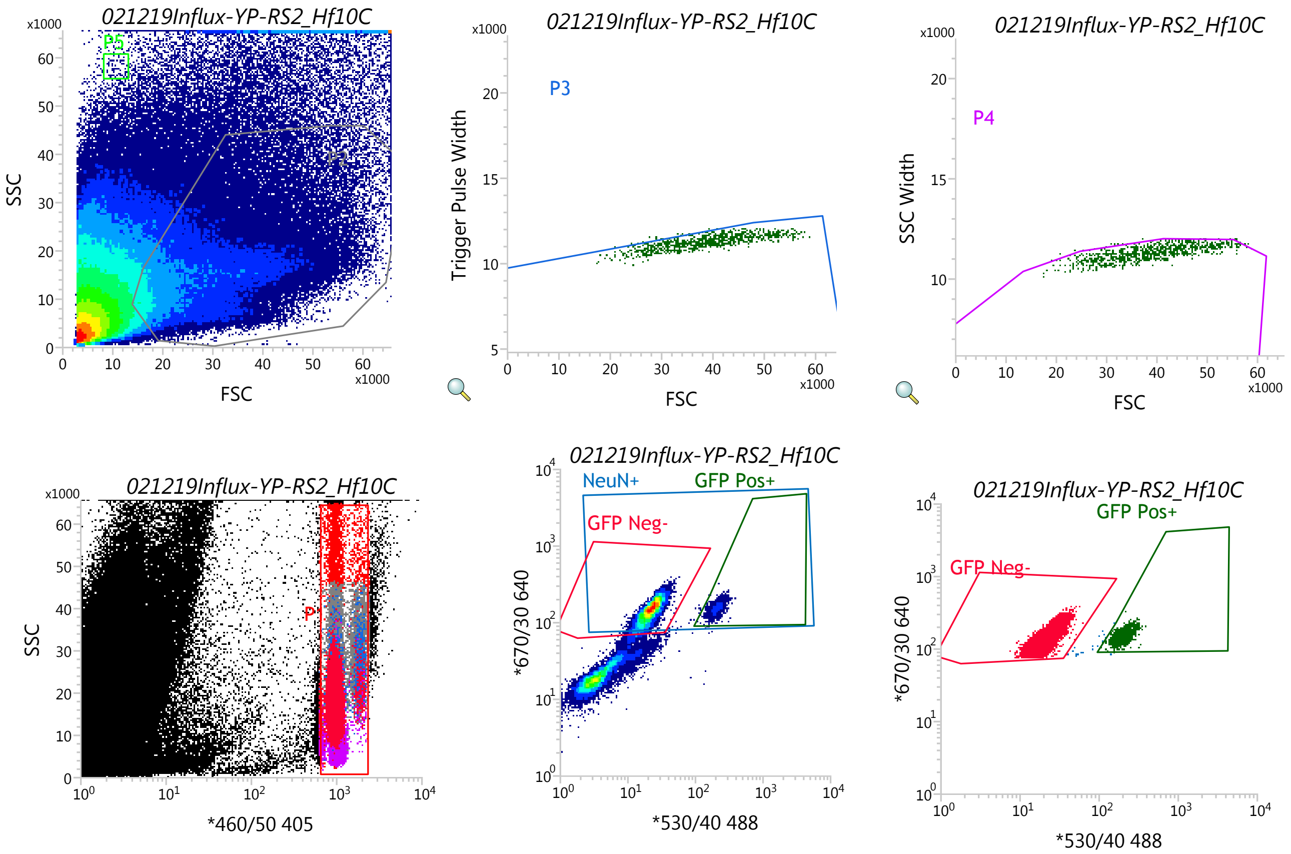

Statistics: *021219Influx-YP-RS2_Hf10C*

Populations	Events	% Total	% Parent
All Events	668,081	100.00%	####
P1	61,375	9.19%	9.19%
P2	58,027	8.69%	94.55%
P3	46,765	7.00%	80.59%
P4	42,792	6.41%	91.50%
NeuN+	24,181	3.62%	56.51%
GFP Pos+	894	0.13%	3.70%
GFP Neg-	23,266	3.48%	96.22%

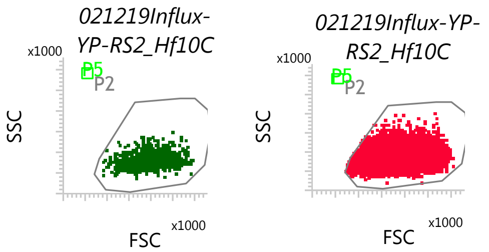

Supplement: Supplementary file 1 — Supplementary Methods: FANS gating. The file shows exemplified gating strategies of FANS in this study. [file 41586_2021_3223_MOESM1_ESM.pdf]
