# Supplementary material for: Epigenomic diversity of cortical projection neurons in the mouse brain
Source: Nature. 2021 Oct 6;598(7879):167–73. doi: 10.1038/s41586-021-03223-w (PMC8494636; doi:10.1038/s41586-021-03223-w)
Supplement: Supplementary file 2 — Reporting Summary [file 41586_2021_3223_MOESM2_ESM.pdf]

nature research

Corresponding author(s): Edward M. Callaway, Joseph R. Ecker

Last updated by author(s): Nov 11, 2020

Reporting Summary

Nature Research wishes to improve the reproducibility of the work that we publish. This form provides structure for consistency and transparency in reporting. For further information on Nature Research policies, see Authors & Referees and the Editorial Policy Checklist.

Statistics

For all statistical analyses, confirm that the following items are present in the figure legend, table legend, main text, or Methods section.

n/a	Confirmed	
☐	☒	The exact sample size (*n*) for each experimental group/condition, given as a discrete number and unit of measurement
☐	☒	A statement on whether measurements were taken from distinct samples or whether the same sample was measured repeatedly
☐	☒	The statistical test(s) used AND whether they are one- or two-sided<br>*Only common tests should be described solely by name; describe more complex techniques in the Methods section.*
☐	☒	A description of all covariates tested
☐	☒	A description of any assumptions or corrections, such as tests of normality and adjustment for multiple comparisons
☐	☒	A full description of the statistical parameters including central tendency (e.g. means) or other basic estimates (e.g. regression coefficient) AND variation (e.g. standard deviation) or associated estimates of uncertainty (e.g. confidence intervals)
☐	☒	For null hypothesis testing, the test statistic (e.g. *F*, *t*, *r*) with confidence intervals, effect sizes, degrees of freedom and *P* value noted<br>*Give P values as exact values whenever suitable.*
☒	☐	For Bayesian analysis, information on the choice of priors and Markov chain Monte Carlo settings
☒	☐	For hierarchical and complex designs, identification of the appropriate level for tests and full reporting of outcomes
☐	☒	Estimates of effect sizes (e.g. Cohen's *d*, Pearson's *r*), indicating how they were calculated

Our web collection on statistics for biologists contains articles on many of the points above.

Software and code

Policy information about availability of computer code

Data collection	BD Influx Sortware v1.2.0.142 (flow cytometry), Freedom EVOware v2.7 (library preparation), Illumina MiSeq control software v3.1.0.13 and NovaSeq 6000 control software v1.6.0/RTA v3.4.4 (sequencing), Olympus cellSens Dimension 1.8 (image acquisition)
Data analysis	FIJI distribution of ImageJ, Scikit-learn 0.20.3, Bedtools 2.27, Scanpy 1.6.0, fimo 5.0.2<br>cemba_data mapping pipeline is available at https://github.com/lhqing/cemba_data.git, including Cutadapt 1.18, Bismark 0.20.0, Bowtie2 2.3.5, Fastqc=0.11, Picard=2.18, Samtools=1.9, Htslib=1.9<br>scanorama: https://github.com/brianhie/scanorama.git<br>Other code are available on https://github.com/zhoujt1994/EpiRetroSeq2020.git

For manuscripts utilizing custom algorithms or software that are central to the research but not yet described in published literature, software must be made available to editors/reviewers. We strongly encourage code deposition in a community repository (e.g. GitHub). See the Nature Research guidelines for submitting code & software for further information.

Data

Policy information about availability of data

All manuscripts must include a data availability statement. This statement should provide the following information, where applicable:

- Accession codes, unique identifiers, or web links for publicly available datasets
- A list of figures that have associated raw data
- A description of any restrictions on data availability

The data analyzed in this study were produced through the Brain Initiative Cell Census Network (BICCN: RRID:SCR_015820) and deposited to NCBI GEO/SRA with accession number GSE150170 and the NEMO Archive (RRID:SCR_002001) under identifier nemo:dat-t2mznz0 accessible at https://assets.nemoarchive.org/dat-t2mznz0. The code for all of the analyses and the link to data browser can be found at https://github.com/zhoujt1994/EpiRetroSeq2020.git

Field-specific reporting

Please select the one below that is the best fit for your research. If you are not sure, read the appropriate sections before making your selection.

☒ Life sciences ☐ Behavioural & social sciences ☐ Ecological, evolutionary & environmental sciences

For a reference copy of the document with all sections, see nature.com/documents/nr-reporting-summary-flat.pdf

Life sciences study design

All studies must disclose on these points even when the disclosure is negative.

Sample size	384 nuclei from each projection (except the MOp→SSp projection from which 768 nuclei were assayed). The sample size allowed us to obtain high coverage methylomes for each projection, and confidently identify differentially methylated genes.
Data exclusions	Poor quality nuclei were excluded from clustering if they failed to meet the following pre-established quality control (QC) thresholds:<br>< 500,000 non-clonal reads<br>> 1% non-conversion rate
Replication	At least 2 male and 2 female mice were injected with AAV-retro-Cre for each projection target. Male and female samples were pooled separately for nuclei preparation. Nuclei collected from the male and female pool were used as biological replicates in the downstream analyses. Methylomes of cells from different replicates are highly similar (Fig. 1e). Results in Fig. 5f and Extended Data Fig. 10b are reproducible in three biological replicates. Results in Fig. 5h and Extended Data Fig. 10c are reproducible in two biological replicates, and each data point represents one replicate.
Randomization	Randomization is not applicable, since the cells collected are random by nature.
Blinding	Blinding is not applicable, since all data are collected from mice.

Reporting for specific materials, systems and methods

We require information from authors about some types of materials, experimental systems and methods used in many studies. Here, indicate whether each material, system or method listed is relevant to your study. If you are not sure if a list item applies to your research, read the appropriate section before selecting a response.

Materials & experimental systems

n/a	Involved in the study	
☐	☒	Antibodies
☒	☐	Eukaryotic cell lines
☒	☐	Palaeontology
☐	☒	Animals and other organisms
☒	☐	Human research participants
☒	☐	Clinical data

Methods

n/a	Involved in the study	
☒	☐	ChIP-seq
☐	☒	Flow cytometry
☒	☐	MRI-based neuroimaging

Antibodies

Antibodies used	anti-GFP antibody, dilution: 1:500, Alexa Fluor 488 (Invitrogen, A-21311)<br>anti-NeuN antibody, dilution: 1:300, EMD Millipore MAB377 conjugated with Alexa Fluor 647 (Invitrogen A20173)
Validation	All antibodies have been previously published for use in immunohistochemistry and flow cytometry experiments. Anti-GFP antibody has been validated in Kim et al. Neuron 2020 (PMID: 32396852).

Animals and other organisms

Policy information about studies involving animals; ARRIVE guidelines recommended for reporting animal research

Laboratory animals	42-49 day old adult male and female INTACT mice (R26R-CAG-loxp-stop-loxp-Sun1-sfGFP-Myc maintained on C57BL/6J background) were used for Epi-Retro-Seq experiments. Adult wildtype C57BL/6J mice were used for double-retrograde labeling experiments. Housing condition: Temperature: 21-23 C, relative humidity: 61-63%.
Wild animals	The study did not involve wild animals.
Field-collected samples	The study did not involve samples collected from the field.

Ethics oversight | All experimental procedures using live animals were approved by the Salk Institute Animal Care and Use Committee.

Note that full information on the approval of the study protocol must also be provided in the manuscript.

Flow Cytometry

Plots

Confirm that:

☒ The axis labels state the marker and fluorochrome used (e.g. CD4-FITC).

☒ The axis scales are clearly visible. Include numbers along axes only for bottom left plot of group (a 'group' is an analysis of identical markers).

☒ All plots are contour plots with outliers or pseudocolor plots.

☒ A numerical value for number of cells or percentage (with statistics) is provided.

Methodology

Sample preparation

Manually dissected mouse brain samples were snap-frozen on dry ice and stored at -80 °C. Prior to nuclei preparation, for each projection, samples from 2 males and 2 females were pooled separately as biological replicates. The frozen brain tissues were transferred to a pre-chiled 2-mL dounce homogenizer with 1 mL ice-cold NIM buffer (0.25M sucrose, 25mM KCl, 5mM MgCl2, 10mM Tris-HCl (pH7.4), 1mM DTT (Sigma 646563), 10µl of protease inhibitor (Sigma P8340)), with 0.1% Triton X-100 and 5µM Hoechst 33342 (Invitrogen H3570), and gently homogenized on ice with the pre-chilled pestle 10-15 times. The homogenate was transferred to pre-chilled microcentrifuge tubes and centrifuged at 1000 rcf for 8 min at 4 °C to pellet the nuclei. The pellet was resuspended in 1 mL ice-cold NIM buffer, and again centrifuged at 1000 rcf for 8 min at 4 °C. The pellet was then resuspended in 450 µL of ice-cold NSB buffer (0.25M sucrose, 5mM MgCl2, 10mM Tris-HCl (pH7.4), 1mM DTT, 9ul of Protease inhibitor), and filtered through 40µM cell strainer. The filtered nuclei suspension was incubated on ice for at least 30 minutes with 50µl of nuclease-free BSA for at least 10 minutes, then incubated with GFP antibody, Alexa Fluor 488 (Invitrogen, A-21311) and anti-NeuN antibody (EMD Millipore MAB377) conjugated with Alexa Fluor 647 (Invitrogen A20173). GFP+/NeuN+ single nuclei were isolated using fluorescence-activated nuclei sorting (FANS) on a BD Influx sorter with 100µm nozzle, and sorted into 384-well plates preloaded with 2µl of digestion buffer for snmC-seq215 (20mL digestion buffer consists of 10mL M-digestion buffer (2×, Zymo D5021-9), 1ml Proteinase K (20mg, Zymo D3001-2-20), 9mL water, and 10µL unmethylated lambda DNA (100pg/µL, Promega, D1521)). The collected plates were incubated at 50 °C for 20 minutes then stored at -20 °C.

Instrument | BD Influx

Software | BD Influx Sortware v1.2.0.142

Cell population abundance | We sorted NeuN-positive and GFP-positive nuclei.

Gating strategy

Intact nuclei were first discriminated from debris by virtue of their bright DNA labeling (Hoechst Height signal) followed by light scattering profiles (Forward Scatter (FSC) Height vs Side Scatter (SSC) Height). Events with high Pulse Width measurements for FSC and SSC were then excluded as aggregates. Next, NeuN-AlexaFluor 647 labelled neuronal nuclei were selected ("*670/30 640" Height) from which GFP positive nuclei were sorted ("*530/40 488" Height).

☒ Tick this box to confirm that a figure exemplifying the gating strategy is provided in the Supplementary Information.
